# Clinical Pharmacokinetic Assessment of Kratom (*Mitragyna speciosa*), a Botanical Product with Opioid-like Effects, in Healthy Adult Participants

**DOI:** 10.3390/pharmaceutics14030620

**Published:** 2022-03-11

**Authors:** Rakshit S. Tanna, James T. Nguyen, Deena L. Hadi, Preston K. Manwill, Laura Flores-Bocanegra, Matthew E. Layton, John R. White, Nadja B. Cech, Nicholas H. Oberlies, Allan E. Rettie, Kenneth E. Thummel, Mary F. Paine

**Affiliations:** 1 Department of Pharmaceutical Sciences, College of Pharmacy and Pharmaceutical Sciences, Washington State University, Spokane, WA 99202, USA; rakshitsanjay.tanna@wsu.edu (R.S.T.); james.nguyen2@wsu.edu (J.T.N.); deena.hadi@wsu.edu (D.L.H.); 2 Center of Excellence for Natural Product Drug Interaction Research, Spokane, WA 99202, USA; nbcech@uncg.edu (N.B.C.); n_oberli@uncg.edu (N.H.O.); rettie@uw.edu (A.E.R.); thummel@uw.edu (K.E.T.); 3 Department of Chemistry and Biochemistry, University of North Carolina at Greensboro, Greensboro, NC 27412, USA; pkmanwill@uncg.edu (P.K.M.); flores.bocanegra.lc@gmail.com (L.F.-B.); 4 Elson S. Floyd College of Medicine, Washington State University, Spokane, WA 99202, USA; layton@wsu.edu; 5 Department of Pharmacotherapy, College of Pharmacy and Pharmaceutical Sciences, Washington State University, Spokane, WA 99202, USA; whitej@wsu.edu; 6 Department of Medicinal Chemistry, School of Pharmacy, University of Washington, Seattle, WA 98195, USA; 7 Department of Pharmaceutics, School of Pharmacy, University of Washington, Seattle, WA 98195, USA

**Keywords:** clinical trials, pharmacokinetics, botanicals, opioids, kratom, compartmental modeling, diastereomers, mitragynine

## Abstract

Increasing use of the botanical kratom to self-manage opioid withdrawal and pain has led to increased kratom-linked overdose deaths. Despite these serious safety concerns, rigorous fundamental pharmacokinetic knowledge of kratom in humans remains lacking. We assessed the pharmacokinetics of a single low dose (2 g) of a well-characterized kratom product administered orally to six healthy participants. Median concentration-time profiles for the kratom alkaloids examined were best described by a two-compartment model with central elimination. Pronounced pharmacokinetic differences between alkaloids with the 3*S* configuration (mitragynine, speciogynine, paynantheine) and alkaloids with the 3*R* configuration (mitraciliatine, speciociliatine, isopaynantheine) were attributed to differences in apparent intercompartmental distribution clearance, volumes of distribution, and clearance. Based on noncompartmental analysis of individual concentration-time profiles, the 3*S* alkaloids exhibited a shorter median time to maximum concentration (1–2 vs. 2.5–4.5 h), lower area under the plasma concentration-time curve (430–490 vs. 794–5120 nM × h), longer terminal half-life (24–45 vs. ~12–18 h), and higher apparent volume of distribution during the terminal phase (960–12,700 vs. ~46–130 L) compared to the 3*R* alkaloids. Follow-up mechanistic in vitro studies suggested differential hepatic/intestinal metabolism, plasma protein binding, blood-to-plasma partitioning, and/or distribution coefficients may explain the pharmacokinetic differences between the two alkaloid types. This first comprehensive pharmacokinetic characterization of kratom alkaloids in humans provides the foundation for further research to establish safety and effectiveness of this emerging botanical product.

## 1. Introduction

The United States was the leading consumer of opioids worldwide between 1990 and 2016 [1]. Hospitalizations and deaths due to opioid overdose have increased exponentially during the last four decades, with the most recent cases attributed to ultrapotent licit and illicit fentanyl products [2]. According to a report from the Centers for Disease Control and Prevention, approximately 48,000 people died of opioid overdose in 2018 alone [3]. Consequently, opioid users are increasingly seeking safer alternatives to pharmaceutical drugs.

**Figure 1:**
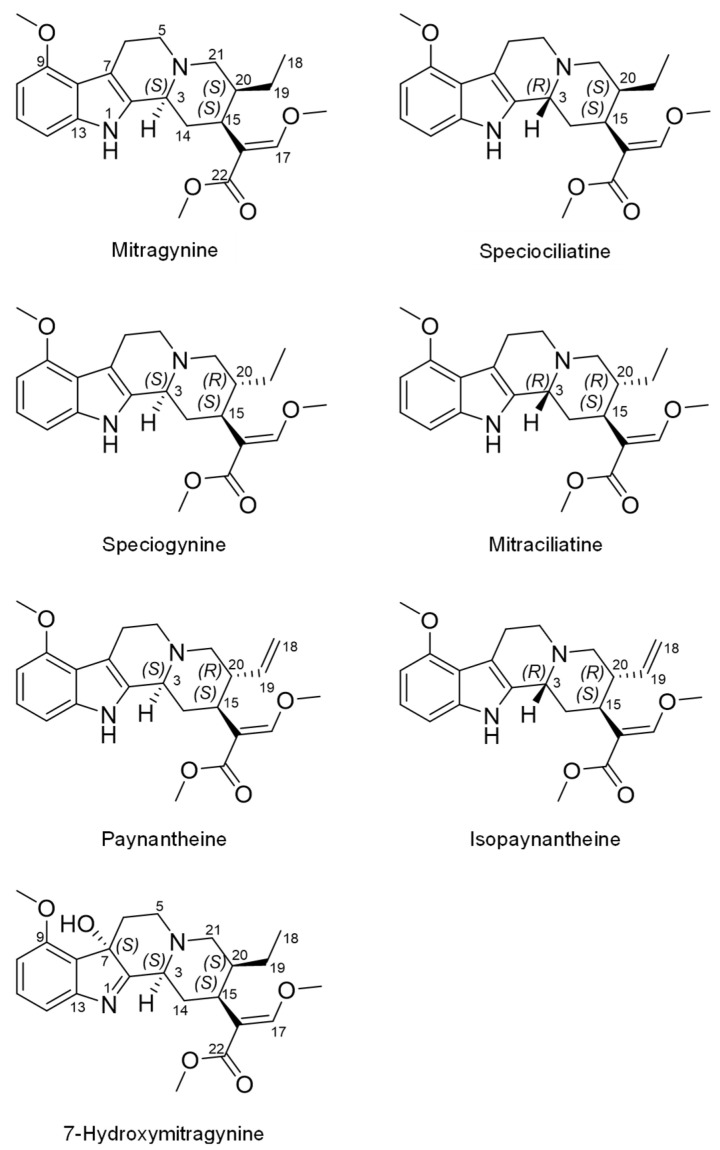
Structures of select indole alkaloids present in the kratom product (K51). Mitragynine, speciogynine, paynantheine, and 7-hydroxymitragynine have the 3*S* configuration, whereas speciociliatine and mitraciliatine have the 3*R* configuration. Mitragynine, speciociliatine, speciogynine, and mitraciliatine are a set of diastereomers. Paynantheine and isopaynantheine are a pair of diastereomers and differ from the other set by having a Δ18(19) double bond. 7-Hydroxymitragynine is a primary active metabolite of mitragynine.

Teas and powders made from the kratom (*Mitragyna speciosa (Korth.) Havil. (Rubiaceae)*) plant are gaining popularity due to their opioid-like effects. Kratom is a tree native to Southeast Asia, where laborers historically consumed leaf preparations to increase stamina and relieve pain [4]. The pain-relieving effects associated with kratom consumption have been attributed to the major alkaloid, mitragynine (Figure 1), as demonstrated in antinociceptive rodent models [5]. Binding affinity assays later showed mitragynine acted as a partial agonist at the human µ-opioid receptor [6].

Besides mitragynine, which constitutes up to 66% of total alkaloid content, at least 50 other structurally related alkaloids have been identified in kratom leaves [7,8]. 7-Hydroxymitragynine, a minor component in the leaf (≤2% of alkaloidal content), is more potent at μ-opioid receptors than mitragynine and exerts stronger opioid-like effects [9]. 7-Hydroxymitragynine is also formed in vivo via cytochrome P450 (CYP)-mediated metabolism of mitragynine [10,11]. Kratom has three other diastereomers of mitragynine with stereocenters at C3, C15, and C20. These include speciogynine (~7% of alkaloidal content), mitraciliatine (<1%), and speciociliatine (<1%). Related phytochemicals include the derivatives paynantheine (~9%) and isopaynantheine (<1%), which have a vinyl group instead of an ethyl group at C20 (Figure 1) [8]. Although knowledge of individual potencies and activities of kratom alkaloids is sparse, they may act synergistically and/or additively to produce the complex pharmacological (opioid and nonopioid) and toxic effects of kratom [6,12,13].

In parallel with the increase in popularity of kratom is an increase in kratom-associated overdose deaths when concomitantly used with other drugs [14]. The United States Food and Drug Administration, Drug Enforcement Administration, and other federal agencies have responded by calling for increased scientific knowledge about the safety of kratom to mitigate this serious public health concern [15,16]. Despite the increasing availability of human in vitro and in vivo animal data [13,17,18], rigorous fundamental information about the pharmacokinetics of kratom alkaloids in humans remains limited.

Only one human pharmacokinetic study with kratom has been reported, which involved 10 healthy male kratom users in Thailand [19]. The participants were administered a kratom tea at varying doses based on mitragynine content (6.25–11.5 mg/day) for seven days. On the eighth day, the tea was again administered at varying doses (mitragynine content, 6.25–23 mg), and plasma and urine were collected from 0–24 h. Mitragynine, which was the only alkaloid measured, displayed a biphasic plasma concentration-time profile. The reported mean (±SD) terminal half-life, 23 ± 16 h, was near or exceeded the sample collection period, raising concern about the robustness of this parameter and subsequently other metrics, including area under the plasma concentration-time curve (AUC), apparent oral clearance (CL/F), renal clearance (CL_R_), and apparent volume of distribution during the terminal phase (V_z_/F).

Based on the aforementioned limitations of prior studies, the objective of the current work was to rigorously characterize the pharmacokinetics of mitragynine and other kratom alkaloids administered as a single fixed dose of a well-characterized kratom product to healthy human adult participants [6,7,20]. The aims were to (1) develop and validate a robust bioanalytical method to quantify the alkaloids in plasma and urine, (2) recover pharmacokinetic parameters for each alkaloid, and (3) conduct follow-up in vitro experiments to gain mechanistic insight into the pharmacokinetic differences among the alkaloids. Our results fill the fundamental knowledge gap regarding the disposition of kratom alkaloids in humans and will aid in the design of future clinical studies to further assess the safety of kratom.

## 2. Materials and Methods

### 2.1. Chemicals and Reagents

Chloroquine, 7-hydroxymitragynine, midazolam, mitragynine, and mitragynine-*d*_3_ were purchased from Cayman Chemical (Ann Arbor, MI, USA). The kratom alkaloids speciogynine, mitraciliatine, speciociliatine, paynantheine, and isopaynantheine were isolated and purified from *Mitragyna speciosa* as detailed previously [7]; all standards were >95% pure as measured by ultraperformance liquid chromatography (UPLC). Alprazolam and *tert*-butyl methyl ether (*t*-BME) were purchased from Sigma-Aldrich (St. Louis, MO, USA). Human liver microsomes (HLMs; H0604, mixed biological sex, pool of 15, lot 1010191) and human intestinal microsomes (HIMs; H0610.I, mixed biological sex, pool of 10, lot 1610314) were purchased from XenoTech, LLC (Kansas City, KS, USA). Acetonitrile and methanol were purchased from Fisher Scientific (Fair Lawn, NJ, USA). Blank human plasma (K_2_EDTA, mixed biological sex, pooled, lot HMN99236) and whole blood (K_2_EDTA, mixed biological sex, pooled, lot HMN541526) were purchased from BioIVT (Westbury, NY, USA). All other chemicals and reagents were analytical grade.

### 2.2. Clinical Pharmacokinetic Study

*Kratom product selection and tea preparation.* Fifty commercial kratom products were sourced from various vendors throughout the United States, and their chemical compositions were compared using untargeted mass spectrometry-based metabolomics [6,7]. Data from the metabolomics study are freely accessible in the Center of Excellence for Natural Product Drug Interaction Research data repository (https://napdicenter.org, accessed on 9 January 2022) [21]. A representative product (described by the manufacturer as yellow Indonesian Micro Powder) was verified to be *Mitragyna speciosa* based on its secondary metabolite profile and DNA barcoding [6]. This product, coded as K51, was selected for the pharmacokinetic study, and a single large batch was acquired. The kratom material was tested further by a third-party laboratory (Covance, Inc., Princeton, NJ, USA) for microbial contamination, pesticides, heavy-metal residue, and solvent residue. The K51 powder was analyzed for kratom alkaloid content expressed as milligrams of alkaloid per gram of dried kratom powder (Table 1) using an established method [6]. Kratom tea was prepared by adding a single low dose of K51 dry leaf powder (2 g) to a 350 mL Styrofoam cup. Hot water (80 °C) (240 mL) was added, and the tea was allowed to steep for three minutes. A sugar packet (4 g) was added to improve palatability. The prepared tea was cooled to 50 °C before administering to the participants.

*Clinical protocol and participants.* The Washington State University (WSU) Institutional Review Board approved the study protocol (IRB #17823) and informed consent form prior to subject recruitment. Study procedures were conducted at the WSU Clinical Research Unit on the Health Sciences Campus under Investigational New Drug status (#145002) and in accordance with the Code of Federal Regulations on the Protection of Human Subjects (45 CFR Part 46). The study was registered with the ClinicalTrials.gov database (NCT04392011). Healthy adults previously exposed to kratom and willing to abstain from kratom use for several weeks were recruited for the study. This strategy allowed characterization of the pharmacokinetic profiles of kratom alkaloids without naloxone (opioid receptor antagonist) administration, which might have confounded the results. Participants provided written informed consent and Health Insurance Portability and Accountability Act authorization prior to screening. Their eligibility to participate in the study was determined based on medical history, physical examination, routine clinical laboratory tests, serum pregnancy tests for female participants of child-bearing potential, and inclusion/exclusion criteria (Table S1). A drug toxicology screen for multiple common drugs of abuse, including several opioids, as well as urine pregnancy tests for female participants, was conducted in the morning of each inpatient visit prior to study procedures.

*Study design*. Three male and four nonpregnant, nonlactating female participants were enrolled. Following an overnight fast, participants presented to the Clinical Research Unit on the morning of their inpatient visit, when vital signs (blood pressure, oxygen saturation, pulse) were recorded. Participants were instructed to drink the entire volume of kratom tea within <10 min, which included rinsing the cup with an additional 100 mL water to ensure consumption of residual tea powder. Serial blood samples (5 mL) were collected into BD K_3_EDTA-containing vacutainer collection tubes (Fisher Scientific Co., Pittsburgh, PA, USA) via an indwelling venous catheter before and at 0.25, 0.5, 0.75, 1.25, 1.75, 2.25, 3.25, 4.25, 6.25, 8.25, and 12.25 h after tea administration (Figure S1). Lunch was provided after the 4.25 h blood draw. Vital signs were recorded periodically throughout the inpatient visit. Participants returned to the Clinical Research Unit for single blood draws at 24, 48, 72, 96, and 120 h after kratom tea administration and recording of vital signs. Upon collection, blood was immediately cooled on ice prior to centrifugation to harvest plasma. Urine was collected from 0–12 h. Upon discharge from the inpatient visit, participants were instructed to collect their urine (12–24 h) until returning for the 24 h blood draw the next morning. Thereafter, subjects collected their urine in 24 h intervals for the next four days, returning each collection at the time of the subsequent outpatient blood draw. Plasma and urine samples were stored at −80 °C and −20 °C, respectively, until bioanalysis.

### 2.3. Bioanalytical Method Development and Validation for Clinical Samples

The UPLC-MS/MS method was developed and validated for simultaneous quantification of kratom alkaloids and related constituents according to United States Food and Drug Administration (FDA) guidelines for the bioanalysis of drugs [22].

*Sample preparation*. Plasma and urine samples were processed using liquid–liquid extraction. In brief, the samples were thawed on ice, briefly vortexed, and a 200 µL aliquot was added to microcentrifuge tubes. Samples were processed on ice to limit the time at ambient temperature and prevent degradation of 7-hydroxymitragynine to mitragynine psuedoindoxyl [23]. Samples were mixed with 5 µL internal standard (mitragynine-*d*_3_, 100 nM), followed by addition of 600 µL *t*-BME. The mixture was vortexed for 10 min and centrifuged at 2270× *g* for 10 min, after which 500 µL of supernatant were transferred to a clean microcentrifuge tube. The supernatant was evaporated to dryness using the Eppendorf™ Vacufuge™ (Brinkmann Instruments, Inc., Westbury, NY, USA) at ambient temperature. The residue was reconstituted with 50 µL methanol (50% *v*/*v*), vortexed for 10 min, and centrifuged at 2270× *g* for 10 min, and the supernatant was subjected to UPLC-MS/MS analysis.

*Instrumentation.* The analytes of interest were quantified using a Shimadzu Nexera X2 UPLC (Shimadzu Corporation, Tokyo, Japan) interfaced with a QTRAP 5500 system (AB Sciex, Framingham, MA, USA) operating in positive electrospray ionization mode. Chromatographic separation was achieved using a reverse-phase column (Acquity UPLC^®^ HSS T3 Column, 1.8 µm, 100 × 2.1 mm) with a VanGuard™ precolumn (Waters, Milford, MA, USA) and heated to 40 °C. A binary gradient consisting of 0.1% formic acid in water (A) and 0.1% formic acid in acetonitrile (B) was operated at a flow rate of 0.5 mL/min. The following gradient was applied: 0–2.0 min, 20% B; 2.0–3.0 min, 20–35% B; 3.0–6.5 min, 35% B; 6.5–7.0 min, 35–95% B; 7.0–8.0 min, 95% B; 8.0–8.1 min, 95–20% B; and 8.1–11.0 min, 20% B. Alkaloid concentrations were quantified by interpolation from matrix-matched calibration curves (0.23–500 nM) prepared using reference standards. The accuracy of all calibration standards and quality controls were within 100% ± 20% at the low limit of quantification or 100% ± 15% above the low limit of quantification.

### 2.4. Pharmacokinetic Analysis

The concentration-time profiles of the seven kratom alkaloids were first evaluated using a compartmental modeling approach within Phoenix WinNonlin (v8.3; Certara, Princeton, NJ, USA). Sparse data points and gradation in the plasma concentrations during the early (“absorption”) phase complicated the robust modeling of individual participant’s concentration-time profiles. As such, median concentration-time profiles were used. Goodness-of-fit was determined based on visual inspection of the plots and other diagnostics, including standard errors of the estimates, Akaike information criterion (AIC), and the Schwarz Bayesian criterion (SBC). Pharmacokinetic parameters are reported as the estimates with standard errors.

The pharmacokinetics of the kratom alkaloids for each participant were next determined using noncompartmental analysis (NCA) methods within Phoenix WinNonlin. Maximum plasma concentration (C_max_) and time to reach C_max_ (t_max_) were obtained directly from the plasma concentration vs. time profiles. The terminal elimination rate constant (λ_z_) was estimated using at least the last three data points of the terminal linear phase of the log-linear concentration-time curve. Samples at the later time points that were below the lower limit of quantitation (LLOQ) were treated as missing data to avoid inappropriate estimation of λ_z_. Terminal half-life (t_1/2_) was calculated as 0.693/λ_z_. The AUC from time zero to the time of the last measurable concentration (AUC_last_) was calculated using the linear-up/log-down trapezoidal method. Total AUC (AUC_inf_) was calculated as the sum of AUC_last_ and C_last_/λ_z_. CL/F was calculated as the ratio of the mass of each alkaloid measured in 2 g of K51 powder to AUC_inf_. Apparent volume of distribution during the terminal phase (V_z_/F) was calculated as (CL/F)/λ_z_. The metabolite-to-parent C_max_ (C_max,m_/C_max,p_) and AUC_inf_ (AUC_inf,m_/AUC_inf,p_) ratios were calculated to compare the relative abundance of 7-hydroxymitragynine to mitragynine in the circulation. Data are reported as medians with ranges.

The cumulative amount of each alkaloid excreted into urine during each collection interval (A_e,interval_) was calculated as the product of the urine concentration and urine volume. The total amount excreted into the urine (A_e,total_) was calculated as the sum of all A_e,interval_ values. The fraction of kratom alkaloid excreted unchanged in the urine (f_e_) was calculated as the ratio of A_e,total_ to the amount of alkaloid measured in 2 g of K51 powder. CL_R_ was calculated as the ratio of A_e,total_ to AUC_last_. Data are reported as medians with ranges.

### 2.5. Follow-Up In Vitro Studies

Metabolic stability in HIMs and HLMs (t_1/2_), unbound fraction in human plasma (f_u,p_) and HLMs (f_u,mic_), and blood-to-plasma concentration ratios (C_B_/C_P_) of kratom alkaloids were determined using experimental techniques described in detail, including the bioanalysis of kratom alkaloids from in vitro samples (Supplementary Materials). Hepatic intrinsic clearance (CL_int,H_) and subsequently hepatic clearance (CL_H,u_) were calculated using the well-stirred model [24] (Supplementary Materials).

## 3. Results

### 3.1. Participants, Safety, and Tolerability of Kratom Tea

Of the eight potential participants screened, seven were enrolled. One female participant was withdrawn immediately from the study due to nausea and subsequent vomiting 20 min after consuming the kratom tea. This participant was replaced with another female participant who experienced nausea and vomiting 2 and 3 h after consuming the tea. She elected to continue her participation but was eventually withdrawn after the 48 h blood collection due to abnormal appearing urine; she was referred to her primary care physician for follow-up. This observation was deemed likely unrelated to kratom consumption. The six participants self-identified as White (one male and three females), Black (one male), or multiracial (one male). Participant age ranged from 26–40 years. None of the participants failed the drug toxicology screen, and all abstained from taking concomitant medications and botanical and other natural products during the entire study period. Except for the two discontinued female participants, the kratom tea was well tolerated, and none of the participants experienced any severe adverse events. Two participants experienced an adverse event during the study unrelated to kratom (lightheadedness upon placement of an intravenous catheter/first blood draw and mild headache, respectively) that did not result in study discontinuation.

### 3.2. Bioanalysis of Kratom Alkaloids in Human Plasma and Urine

**Figure 2:**
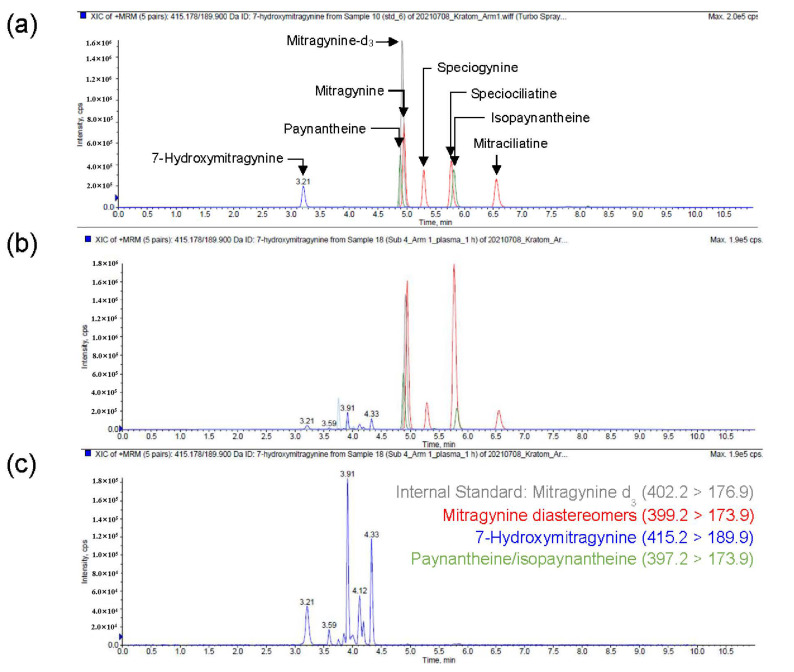
Representative chromatograms for simultaneous quantification of kratom alkaloids in human plasma. (**a**) Total ion chromatogram (TIC) of reference standards depicting separation of alkaloids with the same MRM transitions (diastereomers). (**b**) TIC of a representative human plasma sample 1-h post-kratom tea administration. (**c**) Extracted ion chromatogram (415 > 190) depicting 7-hydroxymitragynine and other products.

A simple, selective, and sensitive UPLC-MS/MS method was developed and validated for simultaneous quantification of kratom alkaloids and related constituents. Alkaloids with the same multiple reaction monitoring (MRM) transitions (diastereomers) were well separated chromatographically to ensure accurate quantification of each without interference. Likewise, multiple hydroxylated products of mitragynine and/or mitragynine diastereomers with the same MRM transition were observed. These products also were separated chromatographically (Figure 2) to confirm and quantify products of interest using available reference standards, including 7-hydroxymitragynine. The attained LLOQ (0.23 nM) enabled accurate recovery of pharmacokinetic parameters for all alkaloids except isopaynantheine and 7-hydroxymitragynine, which were quantifiable up to at least 96 h in plasma of the five participants who completed the study; isopaynantheine and 7-hydroxymitragynine were quantifiable up to 72 and 24 h, respectively. All alkaloids were quantifiable up to 120 h in urine in the five participants who completed the study. All alkaloids were quantifiable up to 48 h in plasma and urine in the one subject who was withdrawn after the 48 h blood collection.

### 3.3. Pharmacokinetics of Kratom Alkaloids

**Figure 3:**
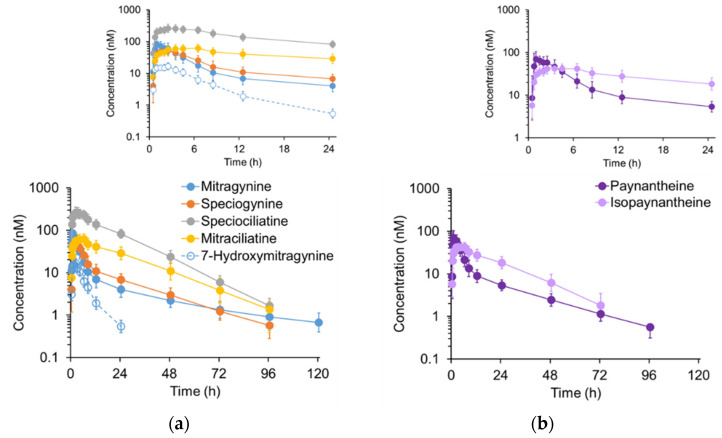
Plasma concentration-time profiles for (**a**) mitragynine, mitragynine diastereomers, and 7-hydroxymitragynine and (**b**) paynantheine and isopaynantheine following oral administration of a well-characterized kratom product as a tea to six participants. Kratom tea was prepared with 2 g of yellow Indonesian Micro Powder (K51) in 240 mL of hot water (80 °C), which was allowed to steep for three minutes. A sugar packet (4 g) was added to improve palatability. The prepared tea was cooled to 50 °C before administration to the participants. Symbols and error bars denote geometric means and 90% confidence intervals, respectively. Insets show the 0–24 h profiles to better visualize alkaloid concentrations during the intensive sampling period after administration of the kratom tea.

Kratom alkaloids with the 3*S* configuration, hereinafter termed 3*S* alkaloids (mitragynine, speciogynine, paynantheine), followed a biphasic concentration-time profile, whereas alkaloids with the 3*R* configuration, hereinafter termed 3*R* alkaloids (mitraciliatine, speciociliatine, isopaynantheine), appeared to follow a monophasic concentration-time profile (Figure 3).

**Figure 4:**
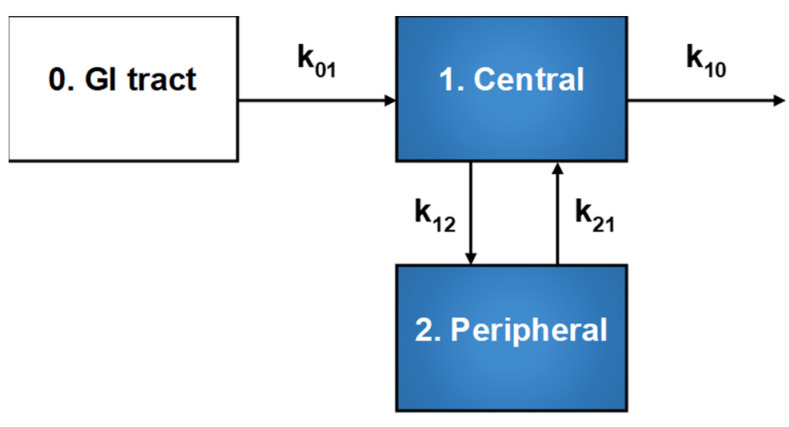
Schematic illustration of the two-compartment model with first-order input and elimination rate used to describe the median concentration-time profiles of all kratom alkaloids. Compartments 1 and 2 represent the central and peripheral compartments, respectively. The model was parameterized with the microconstants k_01_, k_12_, k_21_, and k_10_; k_01_ (or k_abs_) is the rate constant for absorption into the central compartment constant, k_12_ and k_21_ are the intercompartmental distribution rate constants, and k_10_ is the rate constant for loss from the central compartment.

Contrary to visual inspection, compartmental analysis of the median concentration-time profiles (Figure S3) of all the kratom alkaloids revealed that a two-compartment model with first-order input and elimination rate, elimination from the central compartment, and a lag time with a weighting factor of 1/ŷ^2^ best described the data based on goodness-of-fit criteria (Figure 4). The model was parameterized with the microconstants k_01_, k_12_, k_21_, and k_10_; k_01_ (or k_abs_) is the rate constant for absorption into the central compartment, k_12_ and k_21_ are the intercompartmental distribution rate constants, and k_10_ is the elimination rate constant for loss from the central compartment. Compartmental analysis displayed distinct features between the two types of alkaloids, especially in terms of the apparent central (V_1_/F) and peripheral (V_2_/F) volumes of distribution, apparent intercompartmental distributional clearance (CL_D_/F), and CL/F. The 3*S* alkaloids displayed higher V_2_/F, CL_D_/F, and CL/F than the 3*R* alkaloids (Table 2).

Regarding NCA of the individual concentration-time profiles, all extrapolated AUCs for alkaloids beyond the respective C_last_ were less than 20%, ensuring robust estimates of terminal t_1/2_, CL/F, V_z_/F, and AUC_inf_. These outcomes, along with C_max_ and t_max_, were distinct between the two types of alkaloids. That is, the 3*S* alkaloids exhibited a longer terminal t_1/2_, higher CL/F and V_z_/F, lower dose-normalized AUC_inf_ and C_max_, and shorter t_max_ than the 3*R* alkaloids (Table 1). The 7-hydroxymitragynine-to-mitragynine C_max_ and AUC_inf_ ratios ranged from ~0.24–0.27.

**Figure 5:**
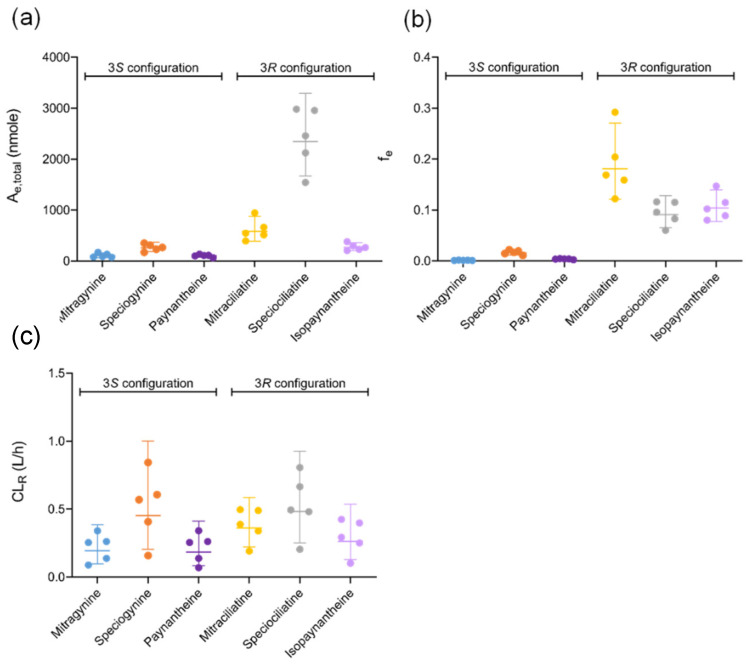
(**a**) Total amount of kratom alkaloids excreted in urine (A_e,total_) after kratom tea administration over a 120 h collection period. (**b**) Fraction of the administered dose excreted unchanged in the urine (f_e_) from 0–120 h. (**c**) Renal clearance (CL_R_) of kratom alkaloids. Symbols denote individual data points for the five participants who completed the study. Horizontal lines denote geometric means. Error bars denote 95% confidence intervals.

Minimal amounts of the kratom alkaloids were excreted unchanged in the urine; the f_e_ for the 3*R* alkaloids was higher than that for the 3*S* alkaloids (Figure 5). CL_R_ was comparable between the two types of alkaloids (Table 1).

### 3.4. Follow-Up In Vitro Studies

**Figure 6:**
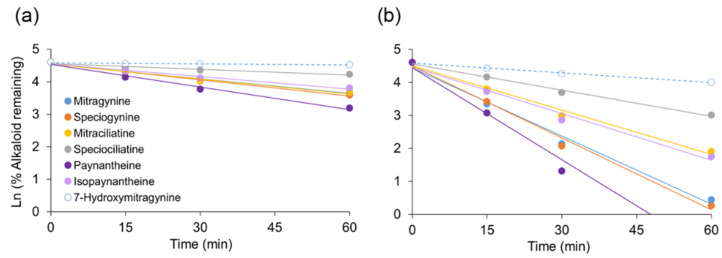
NADPH-dependent depletion of each kratom alkaloid (1 µM) incubated with human (**a**) intestinal and (**b**) liver microsomes (0.5 mg/mL) at 37 °C. Symbols denote means of triplicate incubations, which varied less than 15%. Error bars are not shown for visualization purposes. Lines denote linear regression (R^2^ > 0.9) of the data, and negative slopes indicate in vitro rate of elimination (k_e_). The 60 min timepoint for paynantheine in (**b**) is not shown to allow direct comparison between (**a**,**b**) using a common scale. The k_e_ was estimated using all data points.

All kratom alkaloids except 7-hydroxymitragynine underwent extensive NADPH-dependent depletion in human microsomal incubations (Table 3). The microsomal t_1/2_ for the 3*S* kratom alkaloids was shorter than that for the 3*R* alkaloids. The extent of depletion was higher in HLMs compared to HIMs, but with the same alkaloid rank order (Figure 6). All alkaloids were highly bound to human plasma proteins (f_u,p_ < 0.06) and low to moderately bound to microsomal proteins at 0.5 mg/mL (f_u,mic_, 0.34–0.88). The f_u,p_ and f_u,mic_ for the 3*S* alkaloids were in general higher than the 3*R* alkaloids. The C_B_/C_P_ for all alkaloids was <1, indicating minimal partitioning/binding with red blood cells. A low CL_H,u_ (<5 mL/min/kg) was calculated for all alkaloids using the well-stirred model.

## 4. Discussion

Consumption of oral supplements made from kratom leaves as natural alternatives to manage opioid withdrawal symptoms and pain continues to increase. Deaths linked to the concomitant use of kratom with various pharmaceutical drugs, particularly opioids, have raised safety concerns among United States federal regulators [25]. Currently, regulation of kratom in the United States is ambiguous due to lack of scientific literature addressing safety concerns. Despite increasing publications focused on the complex chemistry and biological effects of kratom alkaloids [6], rigorous fundamental information regarding the pharmacokinetics of these phytochemicals in humans remains lacking. The current work describes the first comprehensive pharmacokinetic characterization of kratom alkaloids in healthy adults administered a single low dose of a well-characterized kratom product (K51) as a tea, the most common route of consumption.

The previous pharmacokinetic study involving 10 adult male participants and a kratom tea included variable doses of mitragynine, quantification of only mitragynine in plasma and urine, and a 0–24 h collection period [19]. The reported terminal t_1/2_ was approximately 24 h, raising concerns about the accuracy of this parameter, as well as the subsequent metrics, including AUC_inf_, CL/F, and V_z_/F. Based on these limitations, the kratom tea used in the current work was thoroughly characterized and prepared in a standardized manner to maintain consistency in alkaloid content, plasma and urine collection was extended to 120 h, and several alkaloids in addition to mitragynine were quantified.

The kratom tea was generally well tolerated, as all the participants were previously exposed to kratom. The nausea and vomiting experienced by two female participants were not unexpected, as both are common side effects of opioids [26]. Although available, use of naloxone was not required to manage these common side effects. No basal plasma concentrations of any alkaloid were detected in the six study participants, ensuring abstinence from kratom before the study. Plasma concentrations of all alkaloids were quantifiable by 15 min after tea administration, suggesting rapid absorption into the systemic circulation. The plasma concentration-time profiles of all alkaloids displayed multiple peaks or gradations during the absorption phase (up to 6 h) after tea administration (Figure S2), which could have reflected delayed gastric emptying common with opioids and/or absorption windows.

Upon visual inspection, plasma concentration-time profiles (Figure 3) of alkaloids with the 3*S* configuration (mitragynine, speciogynine, paynantheine) displayed a biphasic disposition pattern, whereas plasma concentrations of alkaloids with the 3*R* configuration (mitraciliatine, speciociliatine, isopaynantheine) appeared to follow a monophasic disposition pattern. Traditional compartmental analysis methods were used initially to characterize the pharmacokinetics of each kratom alkaloid. However, robust estimates of key parameters could not be obtained for each participant due to sparse data points and gradations during the early (absorptive) phase (Figure S2), precluding assessment of the interindividual variability in the pharmacokinetics of each alkaloid. Therefore, median concentration-time profiles were used to gauge general pharmacokinetic behavior of the two types of alkaloids.

Despite clear visual differences in the pharmacokinetic profiles between the two types of kratom alkaloids, a two-compartment model best described both types. However, the 3*S* alkaloids showed more extensive tissue distribution than the 3*R* alkaloids, as indicated by both a lower V_1_/F relative to V_2_/F (3*S* alkaloids, 157–1170 vs. 468–5620 L; 3*R* alkaloids, 35.5–75.1 vs. 12.8–19.8) and a higher CL_D_/F compared to the 3*R* alkaloids (33.9–213 vs. 1.38–2.59 L/h) (Table 2). The distributional trend observed with mitragynine was consistent with that reported for rats (V_1_, 1.7 L/kg vs. V_2_, 6.3 L/kg) administered mitragynine (5 mg/kg) intravenously [27]. The shorter t_1/2,α_ (1.5–1.78 vs. 2.88–4.42 h) and higher k_12_ (0.166–0.215 vs. 0.0281–0.0728 h*^−^*^1^) recovered for the 3*S* compared to the 3*R* alkaloids indicated more rapid tissue distribution of the 3*S* alkaloids. The longer t_1/2,β_ (21.3–37.3 vs. 14.8–17.5 h) and lower k_21_ (0.0379–0.072 vs. 0.107–0.139 h*^−^*^1^) recovered for the 3*S* compared to the 3*R* alkaloids further indicated a slower redistribution back to the systemic circulation for subsequent elimination. With the exception of speciogynine, the 3*S* alkaloids were absorbed more rapidly than the 3*R* alkaloids (k_01_, 0.970–4.10 vs. 0.706–1.31 h*^−^*^1^). The shorter t_max_ for the 3*S* compared to the 3*R* alkaloids (1.13–2.03 vs. 2.63–3.45 h) further indicated that the 3*S* alkaloids were absorbed, as well as distributed, more rapidly than the 3*R* alkaloids.

NCA methods were used to determine the pharmacokinetics of all alkaloids in each participant, enabling assessment of the variation in alkaloid disposition (Table 1) and comparison with the previous clinical pharmacokinetic study [19]. The previous study reported a terminal t_1/2_ for mitragynine of approximately 24 h, which was roughly half that observed in the current study (45 h). The approximately fourfold higher C_max_ at a similar dose estimated in the previous study compared to the current study (~300 vs. 80 nM) likely reflected accumulation of mitragynine after multiple dosing. Despite a lower abundance of speciogynine in K51 (~15% that of mitragynine), speciogynine C_max_ was approximately two-thirds that of mitragynine, and AUC_last_ and AUC_inf_ were comparable. Paynantheine, also of lower abundance in K51 (30% that of mitragynine), showed similar trends in C_max_ and AUC_inf_ as the other two 3*S* alkaloids. The lower CL/F for speciogynine and paynantheine (15% and 30% of mitragynine CL/F) explains these observations. The dose-normalized C_max_ and AUC_inf_ of the 3*R* alkaloids exceeded those of the 3*S* alkaloids (~60–110 vs. 4.2–16 nM/mg and 1000–1800 vs. 22–150 nM×h/mg, respectively), which can be attributed to both a lower CL/F and lower V_z_/F for the 3*R* compared to the 3*S* alkaloids.

The pronounced differences in pharmacokinetic behavior between the two configurations prompted a series of in vitro studies to determine potential mechanisms for these differences. Specifically, f_u,p_, C_B_/C_P_, and in vitro t_1/2_ were determined for each alkaloid and compared to the apparent distribution and clearance properties. All alkaloids showed high extents of plasma protein binding (f_u,p_ < 0.06) with limited binding to blood components (C_B_/C_P_ < 1.0). However, f_u,p_ values for the 3*S* alkaloids were generally higher than those for the 3*R* alkaloids, suggesting higher unbound plasma concentrations of the 3*S* alkaloids available for tissue distribution. In vitro t_1/2_ obtained from both HIMs and HLMs indicated more rapid metabolism of the 3*S* alkaloids compared the 3*R* alkaloids (Figure 6). A low to moderate CL_H,u_ (Table 3) was predicted for all the alkaloids using the well-stirred model. Although CL_H,u_ for the 3*S* alkaloids was approximately threefold higher than the 3*R* alkaloids, the longer t_1/2,β_ observed for the former suggests that distribution to non-eliminating organs rate-limits systemic plasma and hepatic exposure.

Differences in distribution coefficients were hypothesized to represent another mechanism that may drive the pharmacokinetic differences among the alkaloids given that passive permeability greatly depends on the lipophilicity/partition coefficient. The 3*S* kratom alkaloids were reported to have higher distribution coefficients (C_H_/C_B_), determined using a heptane–buffer (pH 7.4) system, compared to the 3*R* alkaloids (68 and 14.5 for mitragynine and speciogynine, respectively, vs. 4.8 and 4.0 for mitraciliatine and speciociliatine, respectively) [28]. These values reasonably correlated with the distinct tissue distribution parameters (V_2_/F, CL_D_/F, V_z_/F) between the alkaloid types. That is, the 3*S* alkaloids, which had higher distribution coefficients than the 3*R* alkaloids, were more extensively distributed into the tissues (higher V_2_/F and V_z_/F) than the 3*R* alkaloids (Table 1 and Table 2). However, this hypothesis requires further testing, for example, by determining the passive permeability of each alkaloid using an in vitro cell permeability assay and the role of membrane transporters in differential flux of the alkaloids through tissues.

The C_max_ and AUC_inf_ of 7-hydroxymitragynine were approximately 24–27% of corresponding mitragynine values. Given the trace amount of 7-hydroxymitragynine present in K51 (Table S2), this analyte in plasma was assumed to be largely formed from mitragynine in vivo. The two participants in which plasma concentrations were measurable beyond 24 h displayed biphasic concentration-time profiles (Figure S4). The terminal phase of 7-hydroxymitragyinine was reasonably parallel with mitragynine in both participants, further suggesting 7-hydroxymitragynine was eliminated via formation rate-limited kinetics.

All the alkaloids examined were detected in urine up to 120 h after kratom tea administration. The unchanged A_e,total_ of each alkaloid excreted in the urine was proportional to the corresponding AUC_inf_. The f_e_ was higher for the 3*R* alkaloids compared to the 3*S* alkaloids. Regardless of the A_e,total_ and f_e_, CL_R_ for all the kratom alkaloids was much lower than effective renal plasma flow (<0.5 vs. 36 L/h), suggesting a limited contribution by the kidney to overall elimination.

There were limitations to the current work. First, the pharmacokinetics of the kratom alkaloids were assessed after a single low dose of kratom (2 g) administered orally. Extrapolating to higher and/or multiple doses is cautioned due to potential nonlinearities. Second, due to the multiexponential disposition of the alkaloids, the terminal half-lives obtained in this study overestimated accumulation upon multiple dosing. A more appropriate t_1/2_, such as the effective/operational t_1/2_, is needed to better predict systemic alkaloid concentrations with chronic use [29,30]. Third, this study involved a small number of participants. A larger sample size is needed to better characterize the extent of interindividual variability in kratom alkaloid disposition. Fourth, this study characterized the pharmacokinetics of seven key kratom alkaloids. Additional alkaloids and their metabolites that may contribute to the pharmacological effects of kratom are increasingly being identified. Reference standards and robust bioanalytical methods are needed to quantify these other alkaloids and metabolites in biological matrices to advance the understanding of the disposition and action of kratom.

In summary, the current work represents the first comprehensive pharmacokinetic characterization of kratom alkaloids in healthy adult participants administered a single low dose of a well-characterized kratom product administered as a tea. All kratom alkaloids examined displayed biphasic disposition profiles. In general, the 3*S* alkaloids (mitragynine, speciogynine, paynantheine) showed a higher V_2_/F, CL_D_/F, and V_z_/F than the 3*R* alkaloids (mitraciliatine, speciociliatine, isopaynantheine). Differences in distribution and clearance properties may contribute to the distinct pharmacokinetic profiles observed between the two types. These results fill a fundamental knowledge gap and form the foundation for further research to establish the safety and effectiveness of kratom. For example, designing well-informed clinical studies with kratom is now feasible to characterize temporal relationships between systemic kratom alkaloid concentrations and pharmacological effects. Physiologically based pharmacokinetic models of these alkaloids can also be developed and verified to predict potential pharmacokinetic interactions between kratom and pharmaceutical drugs, including drugs of abuse [31]. Such information will enable regulatory agencies to make informed decisions about the safe use of this increasingly popular botanical natural product, thereby addressing ongoing public health concerns.

## Figures and Tables

**Table 1:** Noncompartmental-analysis-derived pharmacokinetics of kratom alkaloids in healthy adult participants (*n* = 5 who completed the study) administered a well-characterized kratom product (2 g) as a tea.

Alkaloid (mg/g Kratom Powder)	Median (Range)			
	Plasma		Urine	
**Mitragynine**	t_1/2_ (h)	45.3 (31.9–50.2)	A_e_ (nmol)	102 (78–134)
(19.48 ± 0.81)	t_max_ (h)	1 (0.75–1.5)	f_e_	0.0010 (0.0008–0.0013)
	C_max_ (nM)	81.9 (50.1–177)	CL_R_ (L/h)	0.194 (0.129–0.291)
	AUC_0–120h_ (nM×h)	388 (300–1240)		
	AUC_inf_ (nM×h)	420 (324–1360)		
	V_z_/F (L)	12,700 (5190–19,700)		
	CL/F (L/h)	233 (71.7–302)		
**Speciogynine**	t_1/2_ (h)	23.5 (16.1–28.3)	A_e_ (nmol)	258 (210–317)
(3.18 ± 0.13)	t_max_ (h)	2 (1–3.5)	f_e_	0.016 (0.013–0.020)
	C_max_ (nM)	51.4 (34.2–121)	CL_R_ (L/h)	0.451 (0.282–0.723)
	AUC_0–120h_ (nM×h)	469 (368–1080)		
	AUC_inf_ (nM×h)	477 (379–1120)		
	V_z_/F (L)	962 (584–1235)		
	CL/F (L/h)	33.5 (14.3–42.1)		
**Mitraciliatine**	t_1/2_ (h)	17.8 (11.2–24.7)	A_e_ (nmol)	586 (461–744)
(0.647 ± 0.035)	t_max_ (h)	4.5 (3.5–6.5)	f_e_	0.18 (0.14–0.23)
	C_max_ (nM)	73.5 (34.9–98.6)	CL_R_ (L/h)	0.361 (0.271–0.481)
	AUC_0–120h_ (nM×h)	1160 (1030–3460)		
	AUC_inf_ (nM×h)	1160 (1040–3520)		
	V_z_/F (L)	46.0 (26.2–74.0)		
	CL/F (L/h)	2.78 (0.92–3.11)		
**Speciociliatine**	t_1/2_ (h)	12.3 (10.4–21.1)	A_e_ (nmol)	2350 (1920–2870)
(5.12 ± 0.26)	t_max_ (h)	2.5 (1–3.5)	f_e_	0.091 (0.075–0.11)
	C_max_ (nM)	308 (154–380)	CL_R_ (L/h)	0.482 (0.327–0.709)
	AUC_0–120h_ (nM×h)	5110 (3190–7550)		
	AUC_inf_ (nM×h)	5120 (3200–7560)		
	V_z_/F (L)	130 (60.1–159)		
	CL/F (L/h)	5.01 (3.40–8.04)		
**Paynantheine**	t_1/2_ (h)	27.0 (17.7–30.8)	A_e_ (nmol)	101 (81.9–124)
(5.86 ± 0.26)	t_max_ (h)	1 (0.75–2.5)	f_e_	0.0034 (0.0028–0.0042)
	C_max_ (nM)	61.1 (56.4–157)	CL_R_ (L/h)	0.185 (0.115–0.296)
	AUC_0–120h_ (nM×h)	428 (383–917)		
	AUC_inf_ (nM×h)	438 (389–956)		
	V_z_/F (L)	1940 (1370–2620)		
	CL/F (L/h)	67.4 (30.9–76.0)		
**Isopaynantheine**	t_1/2_ (h)	14.4 (11.8–20.9)	A_e_ (nmol)	269 (226–320)
(0.512 ± 0.010)	t_max_ (h)	4.5 (2.5–6.5)	f_e_	0.10 (0.087–0.12)
	C_max_ (nM)	48.8 (26.2–68.2)	CL_R_ (L/h)	0.262 (0.172–0.401)
	AUC_0–120h_ (nM×h)	784 (662–2040)		
	AUC_inf_ (nM×h)	794 (667–2130)		
	V_z_/F (L)	55.5 (36.6–76.0)		
	CL/F (L/h)	3.25 (1.21–3.87)		
**7-Hydroxymitragynine**	t_1/2_ (h)	5.67 (5.03–6.52)	A_e_ (nmol) ^a^	179 (120–268)
(< LOQ)	t_max_ (h)	1 (0.75–2.5)	f_e_	NA
	C_max_ (nM)	16.1 (11.9–22.2)	CL_R_ (L/h)	2.03 (1.57–2.63)
	AUC_0–120h_ (nM×h)	103 (57.5–120)		
	AUC_inf_ (nM×h)	106 (60.8–126)		
	C_max,m_/C_max,p_	0.27 (0.07–0.28)		
	AUC_inf,m_/AUC_inf,p_	0.24 (0.07–0.29)		

t_1/2_, terminal half-life; C_max_, maximum plasma concentration; t_max_, time to reach C_max_; AUC_0–120h_, area under the plasma-concentration time curve from time zero to 120 h; AUC_inf_, area under the plasma-concentration time curve from time zero to time infinity; V_z_/F, apparent volume of distribution during the terminal phase; CL/F, oral clearance; A_e,0–120h_, cumulative amount excreted unchanged in the urine from time zero to 120 h; f_e_, fraction of amount of kratom alkaloid measured in 2 g of K51 powder excreted unchanged in the urine; CL_R_, renal clearance; C_max,m_/C_max,p_, 7-hydroxymitragynine-to-mitragynine C_max_ ratio; AUC_inf,m_/AUC_inf,p_, 7-hydroxymitragynine-to-mitragynine AUC_inf_ ratio; ^a^ calculated based on unhydrolyzed urine data; LOQ, limit of quantification; NA, not applicable.

**Table 2:** Compartmental model-derived pharmacokinetic estimates of kratom alkaloids in healthy adult participants (*n* = 5 who completed the study) administered a well-characterized kratom product (2 g) as a tea.

Metric	Mitragynine	Speciogynine	Paynantheine	Mitraciliatine	Speciociliatine	Isopaynantheine
	Estimate (Standard Error)					
**V_1_/F (L)**	1170 (105)	157 (43.6)	329 (37.2)	35.5 (15.9)	75.1 (10.2)	47.8 (8.41)
**k_01_ (1/h)**	4.10 (1.16)	0.970 (0.352)	2.69 (0.691)	0.706 (0.4)	1.31 (0.321)	1.17 (0.341)
**CL/F (L/h)**	227 (8.11)	32.7 (1.38)	62.9 (2.54)	2.44 (0.136)	5.16 (0.233)	3.07 (0.154)
**V_2_/F (L)**	5620 (524)	468 (42.9)	895 (79.1)	18.6 (14.5)	19.8 (10.7)	12.8 (8.28)
**CL_D_/F (L/h)**	213 (20.7)	33.9 (5.93)	54.7 (7.63)	2.59 (3.73)	2.11 (2.18)	1.38 (1.96)
**t_lag_ (h)**	0.49 (0.004)	0.48 (0.004)	0.49 (0.004)	0.42 (0.018)	0.45 (0.010)	0.45 (0.011)
**AUC (nM** **×** **h)**	431 (15.4)	488 (20.6)	470 (19)	1320 (73.7)	4980 (225)	842 (42.3)
**t_1/2,α_ (h)**	1.76 (0.163)	1.5 (0.47)	1.78 (0.244)	2.88 (3.13)	4.42 (2.72)	4.41 (4.17)
**t_1/2,β_ (h)**	37.3 (3.32)	21.3 (1.58)	23 (1.85)	17.5 (1.43)	14.8 (1.13)	15.7 (2.52)
**k_10_ (1/h)**	0.194 (0.0156)	0.208 (0.055)	0.192 (0.0197)	0.0688 (0.0308)	0.0686 (0.001)	0.0642 (0.0119)
**k_12_ (1/h)**	0.182 (0.0228)	0.215 (0.0871)	0.166 (0.033)	0.0728 (0.136)	0.0281 (0.0323)	0.0289 (0.0455)
**k_21_ (1/h)**	0.0379 (0.0045)	0.072 (0.0109)	0.0611 (0.0085)	0.139 (0.106)	0.107 (0.0613)	0.108 (0.103)
**t_max_ (h)**	1.13 (0.111)	2.03 (0.143)	1.36 (0.121)	3.45 (0.335)	2.63 (0.23)	2.84 (0.267)
**C_max_ (nM)**	65.6 (4.22)	53.8 (3.28)	66.2 (4.37)	62.2 (4.96)	279 (17.1)	43.6 (3.05)

V_1_/F, apparent central volume of distribution; k_01_, rate constant for absorption into the central compartment; CL/F, oral clearance; V_2_/F, apparent peripheral volume of distribution; CL_D_/F, apparent intercompartmental distributional clearance; t_lag_, lag time; AUC, area under the plasma concentration-time profile; t_1/2,α_, half-life of early (“distribution”) phase; t_1/2,β_, half-life of terminal phase; k_10_, elimination rate constant for loss from the central compartment; k_12_, intercompartmental distribution rate constant from the central to the peripheral compartment; k_21_, intercompartmental distribution rate constant from the peripheral to the central compartment; t_max_, time to reach C_max_; C_max_, maximum plasma concentration.

**Table 3:** In vitro metabolism, protein binding, and blood-to-plasma concentration ratio data for kratom alkaloids.

Alkaloid	t_1/2,HIMs_ (min)	t_1/2,HLMs_ (min)	f_u,p_	f_u,mic_	C_B_/C_P_	CL_int,H_ (mL/min/kg)	CL_H,u_ (mL/min/kg)
Mitragynine	45.9 ± 0.8	10.1 ± 0.2	0.039 ± 0.003	0.536 ± 0.003	0.93 ± 0.02	31.0	1.22
Speciogynine	41.7 ± 1.3	9.6 ± 0.1	0.057 ± 0.001	0.602 ± 0.009	0.65 ± 0.02	32.6	2.53
Mitraciliatine	45.6 ± 3.4	15.5 ± 0.2	0.019 ± 0.003	0.337 ± 0.006	1.05 ± 0.01	20.2	0.49
Speciociliatine	>60	26.2 ± 0.4	0.040 ± 0.003	0.509 ± 0.012	0.74 ± 0.04	11.9	0.61
Paynantheine	29.9 ± 0.3	7.5 ± 0.3	0.055 ± 0.005	0.516 ± 0.016	0.75 ± 0.01	41.9	2.67
Isopaynantheine	53.5 ± 2.9	14.7 ± 0.2	0.024 ± 0.002	0.412 ± 0.004	0.66 ± 0.02	21.2	0.73

Values represent means ± S.D. of triplicate determinations. t_1/2_, in vitro half-life; HIMs, human intestinal microsomes; HLMs, human liver microsomes; f_u,p_, unbound fraction in plasma; f_u,mic_, unbound fraction in microsomal incubation; C_B_/C_P_, blood-to-plasma concentration ratio; CL_int,H_, in vitro hepatic intrinsic clearance; CL_H,u_, predicted unbound hepatic clearance using the well-stirred model (see Supplemental Materials for details).

## Data Availability

Not applicable.

## Supplementary Materials

The following supporting information can be downloaded at: https://www.mdpi.com/article/10.3390/pharmaceutics14030620/s1, Figure S1: Clinical study design, Figure S2: Representative concentration-time profiles from 0–12 h for each kratom alkaloid in one participant, showing multiple peaks or gradations, Figure S3: Compartmental analysis of the median alkaloid concentration-time profiles, Figure S4: Biphasic concentration-time profile for 7-hydroxymitragynine observed in two participants, Table S1: Clinical study inclusion and exclusion criteria, Table S2: Alkaloid content in commercial kratom product (K51), and supplemental text [32].

## References

[1] Degenhardt L., Grebely J., Stone J., Hickman M., Vickerman P., Marshall B.D., Bruneau J., Altice F.L., Henderson G., Rahimi-Movaghar A. (2019). Global patterns of opioid use and dependence: Harms to populations, interventions, and future action. Lancet.

[2] Hall W., Degenhardt L., Hickman M. (2020). Generational trends in US opioid-overdose deaths. Nat. Med..

[3] Wilson N., Kariisa M., Seth P., Smith IV H., Davis N.L. (2020). Drug and opioid-involved overdose deaths—United States, 2017–2018. Morb. Mortal. Wkly. Rep..

[4] Assanangkornchai S., Muekthong A., Sam-Angsri N., Pattanasattayawong U. (2007). The use of Mitragynine speciosa (“Krathom”), an addictive plant, in Thailand. Subst. Use Misuse.

[5] Matsumoto K., Mizowaki M., Suchitra T., Takayama H., Sakai S.-I., Aimi N., Watanabe H. (1996). Antinociceptive action of mitragynine in mice: Evidence for the involvement of supraspinal opioid receptors. Life Sci..

[6] Todd D., Kellogg J., Wallace E., Khin M., Flores-Bocanegra L., Tanna R., McIntosh S., Raja H., Graf T., Hemby S. (2020). Chemical composition and biological effects of kratom (Mitragyna speciosa): In vitro studies with implications for efficacy and drug interactions. Sci. Rep..

[7] Flores-Bocanegra L., Raja H.A., Graf T.N., Augustinović M., Wallace E.D., Hematian S., Kellogg J.J., Todd D.A., Cech N.B., Oberlies N.H. (2020). The chemistry of kratom [*Mitragyna speciosa*]: Updated characterization data and methods to elucidate indole and oxindole alkaloids. J. Nat. Prod..

[8] Hassan Z., Muzaimi M., Navaratnam V., Yusoff N.H., Suhaimi F.W., Vadivelu R., Vicknasingam B.K., Amato D., von Hörsten S., Ismail N.I. (2013). From Kratom to mitragynine and its derivatives: Physiological and behavioural effects related to use, abuse, and addiction. Neurosci. Biobehav. Rev..

[9] Matsumoto K., Horie S., Ishikawa H., Takayama H., Aimi N., Ponglux D., Watanabe K. (2004). Antinociceptive effect of 7-hydroxymitragynine in mice: Discovery of an orally active opioid analgesic from the Thai medicinal herb Mitragyna speciosa. Life Sci..

[10] Kamble S.H., Sharma A., King T.I., León F., McCurdy C.R., Avery B.A. (2019). Metabolite profiling and identification of enzymes responsible for the metabolism of mitragynine, the major alkaloid of Mitragyna speciosa (kratom). Xenobiotica.

[11] Kruegel A.C., Uprety R., Grinnell S.G., Langreck C., Pekarskaya E.A., Le Rouzic V., Ansonoff M., Gassaway M.M., Pintar J.E., Pasternak G.W. (2019). 7-Hydroxymitragynine is an active metabolite of mitragynine and a key mediator of its analgesic effects. ACS Cent. Sci..

[12] Takayama H. (2004). Chemistry and pharmacology of analgesic indole alkaloids from the rubiaceous plant, Mitragyna speciosa. Chem. Pharm. Bull..

[13] Obeng S., Kamble S.H., Reeves M.E., Restrepo L.F., Patel A., Behnke M., Chear N.J.-Y., Ramanathan S., Sharma A., León F. (2019). Investigation of the adrenergic and opioid binding affinities, metabolic stability, plasma protein binding properties, and functional effects of selected indole-based kratom alkaloids. J. Med. Chem..

[14] Olsen E.O.M., O’Donnell J., Mattson C.L., Schier J.G., Wilson N. (2019). Notes from the field: Unintentional drug overdose deaths with kratom detected—27 states, July 2016–December 2017. Morb. Mortal. Wkly. Rep..

[15] Drug Enforcement Administration (2016). DEA Announces Intent to Schedule Kratom. https://www.dea.gov/press-releases/2016/08/30/dea-announces-intent-schedule-kratom.

[16] (2019). FDA and Kratom. U.S. Food and Drug Administration. https://www.fda.gov/news-events/public-health-focus/fda-and-kratom.

[17] Kamble S.H., Berthold E.C., King T.I., Raju Kanumuri S.R., Popa R., Herting J.R., León F., Sharma A., McMahon L.R., Avery B.A. (2021). Pharmacokinetics of eleven kratom alkaloids following an oral dose of either traditional or commercial kratom products in rats. J. Nat. Prod..

[18] Maxwell E.A., King T.I., Kamble S.H., Raju K.S.R., Berthold E.C., León F., Avery B.A., McMahon L.R., McCurdy C.R., Sharma A. (2020). Pharmacokinetics and safety of mitragynine in beagle dogs. Planta Med..

[19] Trakulsrichai S., Sathirakul K., Auparakkitanon S., Krongvorakul J., Sueajai J., Noumjad N., Sukasem C., Wananukul W. (2015). Pharmacokinetics of mitragynine in man. Drug Des. Dev. Ther..

[20] Kellogg J.J., Paine M.F., McCune J.S., Oberlies N.H., Cech N.B. (2019). Selection and characterization of botanical natural products for research studies: A NaPDI center recommended approach. Nat. Prod. Rep..

[21] Birer-Williams C., Gufford B.T., Chou E., Alilio M., VanAlstine S., Morley R.E., McCune J.S., Paine M.F., Boyce R.D. (2020). A new data repository for pharmacokinetic natural product-drug interactions: From chemical characterization to clinical studies. Drug Metab. Dispos..

[22] (2018). Bioanalytical Method Validation Guidance for Industry.

[23] Kamble S.H., León F., King T.I., Berthold E.C., Lopera-Londoño C., Siva Rama Raju K., Hampson A.J., Sharma A., Avery B.A., McMahon L.R. (2020). Metabolism of a kratom alkaloid metabolite in human plasma increases its opioid potency and efficacy. ACS Pharmacol. Transl. Sci..

[24] Yang J., Jamei M., Yeo K.R., Rostami-Hodjegan A., Tucker G.T. (2007). Misuse of the well-stirred model of hepatic drug clearance. Drug Metab. Dispos..

[25] (2018). Statement from FDA Commissioner Scott Gottlieb, M.D., on the Agency’s Scientific Evidence on the Presence of Opioid Compounds in Kratom, Underscoring Its Potential for Abuse. https://www.fda.gov/news-events/press-announcements/statement-fda-commissioner-scott-gottlieb-md-agencys-scientific-evidence-presence-opioid-compounds.

[26] Ricardo Buenaventura M., Rajive Adlaka M., Nalini Sehgal M. (2008). Opioid complications and side effects. Pain Physician.

[27] Avery B.A., Boddu S.P., Sharma A., Furr E.B., Leon F., Cutler S.J., McCurdy C.R. (2019). Comparative pharmacokinetics of mitragynine after oral administration of Mitragyna speciosa (Kratom) leaf extracts in rats. Planta Med..

[28] Beckett A., Dwuma-Badu D. (1969). The influence of stereochemistry on pKa, rate of quaternization and partition coefficients of corynantheidine-type alkaloids. J. Pharm. Pharmacol..

[29] Boxenbaum H., Battle M. (1995). Effective half-life in clinical pharmacology. J. Clin. Pharmacol..

[30] Sahin S., Benet L.Z. (2008). The operational multiple dosing half-life: A key to defining drug accumulation in patients and to designing extended release dosage forms. Pharm. Res..

[31] Tanna R.S., Tian D.-D., Cech N.B., Oberlies N.H., Rettie A.E., Thummel K.E., Paine M.F. (2021). Refined prediction of pharmacokinetic kratom-drug interactions: Time-dependent inhibition considerations. J. Pharmacol. Exp. Ther..

[32] Thompson M., Ellison S.L., Wood R. (2002). Harmonized Guidelines for Single-Laboratory Validation of Methods of Analysis (IUPAC Technical Report). Pure Appl. Chem..

