# Supplementary material for: Clinical Pharmacokinetic Assessment of Kratom (Mitragyna speciosa), a Botanical Product with Opioid-like Effects, in Healthy Adult Participants
Source: Pharmaceutics. 2022 Mar 11;14(3):620. doi: 10.3390/pharmaceutics14030620 (PMC8950611; doi:10.3390/pharmaceutics14030620)
Supplement: Supplementary file 1 [file pharmaceutics-14-00620-s001.zip › pharmaceutics-1612566-supplementary.pdf]

pharmaceutics

Clinical Pharmacokinetic Assessment of Kratom (*Mitragyna speciosa*), a Botanical Product with Opioid-Like Effects, in Healthy Adult Participants

Rakshit S. Tanna, James T. Nguyen, Deena L. Hadi, Preston K. Manwill, Laura Flores-Bocanegra, Matthew E. Layton, John R. White, Nadja B. Cech, Nicholas H. Oberlies, Allan E. Rettie, Kenneth E. Thummel, and Mary F. Paine

Follow-up in vitro studies.

Metabolic stability of kratom alkaloids in human intestinal and liver microsomes. The NADPH-dependent metabolism of the kratom alkaloids was determined by incubating each alkaloid (final concentration, 1 μM) at 37°C in 96-well plates with HIMs or HLMs (0.5 mg/mL) and NADPH (final concentration, 1 mM) in potassium phosphate buffer (0.1 M, pH 7.4); the final organic (methanol) concentration (v/v) and incubation volume was <0.5% and 400 μL, respectively. Aliquots (50 μL) were removed at 0, 15, 30, and 60 min and quenched with two volumes of ice-cold methanol containing internal standard (alprazolam, 100 nM). The precipitated incubation mixtures were centrifuged at 2270 x g for 10 min, and the supernatants were analyzed for respective alkaloids by UPLC-MS/MS (see below). The in vitro half-life ($t_{1/2} = 0.693/k_e$) was calculated from the first-order elimination rate constant (k_e) obtained from the plot of the natural logarithm of percent alkaloid remaining vs. time. Hepatic intrinsic clearance (CL_{int}) was calculated by scaling k_e with the microsomal content in the liver (45 mg microsomal protein/g of liver) and liver weight (20 g/kg body weight). Hepatic clearance (CL_H) was calculated using the well-stirred model (**eq. 1**), incorporating CL_{int}, fraction unbound in plasma ($f_{u,p}$) and microsomal incubations ($f_{u,mic}$), and blood-to-plasma concentration ratio (C_B/C_P). Unbound hepatic intrinsic clearance ($CL_{int,H,u}$) was calculated as $CL_{int}/f_{u,mic}$, and hepatic blood flow ($Q_{H,B}$) was considered to be 21 mL/min/kg.

$$CL_H = \frac{Q_{H,B} \times f_{u,p} \times CL_{int,H,u}}{Q_{H,B} + f_{u,p} \times CL_{int,H,u}/(C_B/C_P)} \quad (1)$$

Unbound fraction of kratom alkaloids in human plasma and liver microsomes. The $f_{u,p}$ and $f_{u,mic}$ of kratom alkaloids was determined using the equilibrium dialysis method. In brief, each alkaloid (final concentration, 1 μM) was mixed with pooled human plasma or HLMs (0.05 mg/mL) and added to the donor compartment of the 96-well equilibrium dialysis apparatus (HT dialysis LLC, Gales Ferry, CT). An equal volume (150 μL) of potassium phosphate buffer (100 mM) was added to the receiver compartment, which was separated from the donor compartment by a 12-14 kDa semi-permeable membrane (HT dialysis LLC, Gales Ferry, CT). Mixtures were incubated in a 37°C incubator on a rotating platform at 95% relative humidity and 5% CO_2. An aliquot (50 μL) was removed 6 h post-equilibration from each compartment and mixed with an equal volume of opposite matrix (buffer or plasma) before quenching with ice-cold methanol containing internal standard (mitragynine-d_3, 0.1 μM). The precipitated plasma mixtures were centrifuged at 2270 x g for 10 min, and the supernatants were analyzed for kratom alkaloids by UPLC-MS/MS (see below). $f_{u,p}$ and $f_{u,mic}$ was calculated by dividing the concentration of the alkaloid in the receiver compartment to that in the donor compartment at 6 h.

Blood to plasma concentration ratios of kratom alkaloids. Each alkaloid (final concentration, 1 μM) was incubated with pooled human blood in a 37°C incubator on a rotating platform at 95% relative humidity and 5% CO_2. Aliquots of whole blood (200 μL) were removed at 0, 30, 60, and 120 min to assess attainment of equilibrium. Portions (50 μL) of

the removed whole blood were hemolyzed (for red blood cells) in water and proteins precipitated with ice-cold acetonitrile containing internal standard. The remaining whole blood was centrifuged at 1500× *g* for 10 min to harvest plasma. Proteins in the obtained plasma was precipitated with ice-cold acetonitrile containing internal standard. The precipitated blood and plasma mixtures were centrifuged at 2270 x g for 10 min, and the supernatants were analyzed for respective alkaloids by UPLC-MS/MS (see below). C_B/C_P was calculated by dividing the concentration of the alkaloid in blood to that in plasma.

Bioanalysis of kratom alkaloids from in vitro samples. Samples were analyzed for kratom alkaloids using a similar UPLC-MS/MS as described above for human plasma and urine. A reverse-phase column (AQUASIL C18 column, 3 µm, 50 x 2.1 mm; Thermo Scientific, Waltham, MA) with a guard column, heated to 40°C, and a binary gradient consisting of 0.1% formic acid in water (A) and 0.1% formic acid in methanol (B) was used to reduce the run time. The following gradient was applied: 0-0.4 minutes, 10% B; 0.4-1.0 minutes, 10%-95% B; 1.0-2.0 minutes, 95% B; 2.0-2.1 minutes, 95%-10% B; and 2.1-3.0 minutes, 10% B. Kratom alkaloids were quantified by interpolation from matrix-matched calibration curves (1.37-1000 nM) prepared using authentic standards. The accuracy of all the calibration standards and quality controls were within 100% ± 20% at the LLOQ or 100% ± 15% above the LLOQ.

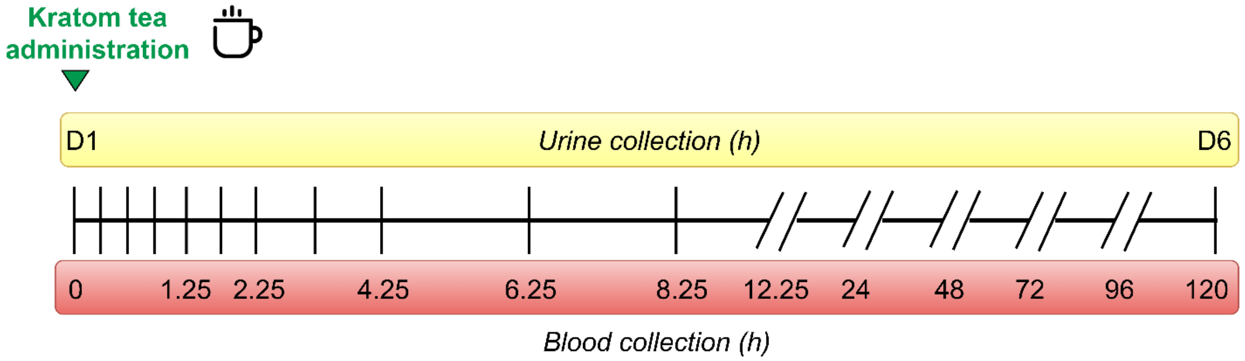

Figure S1. *Clinical study design*. Healthy adult volunteers (3 males, 3 females) were instructed to drink 240 mL of kratom tea, prepared with 2 g of Yellow Indonesian Micro Powder (K51), within 10 min. The tea was prepared by adding 240 mL hot water (80°C) to a 350-mL Styrofoam cup containing 2 g K51 powder; the tea was allowed to steep for at least three minutes. A sugar packet (4 g) was added to improve palatability. The prepared tea was cooled to 50°C before administration to the participants. The cup was rinsed with ~100 mL water, and the participant was instructed to drink this residual. Blood and urine were collected at regular intervals just before kratom tea administration on day 1 (D1) through day 6 (D6) for a total time of 120 hours.

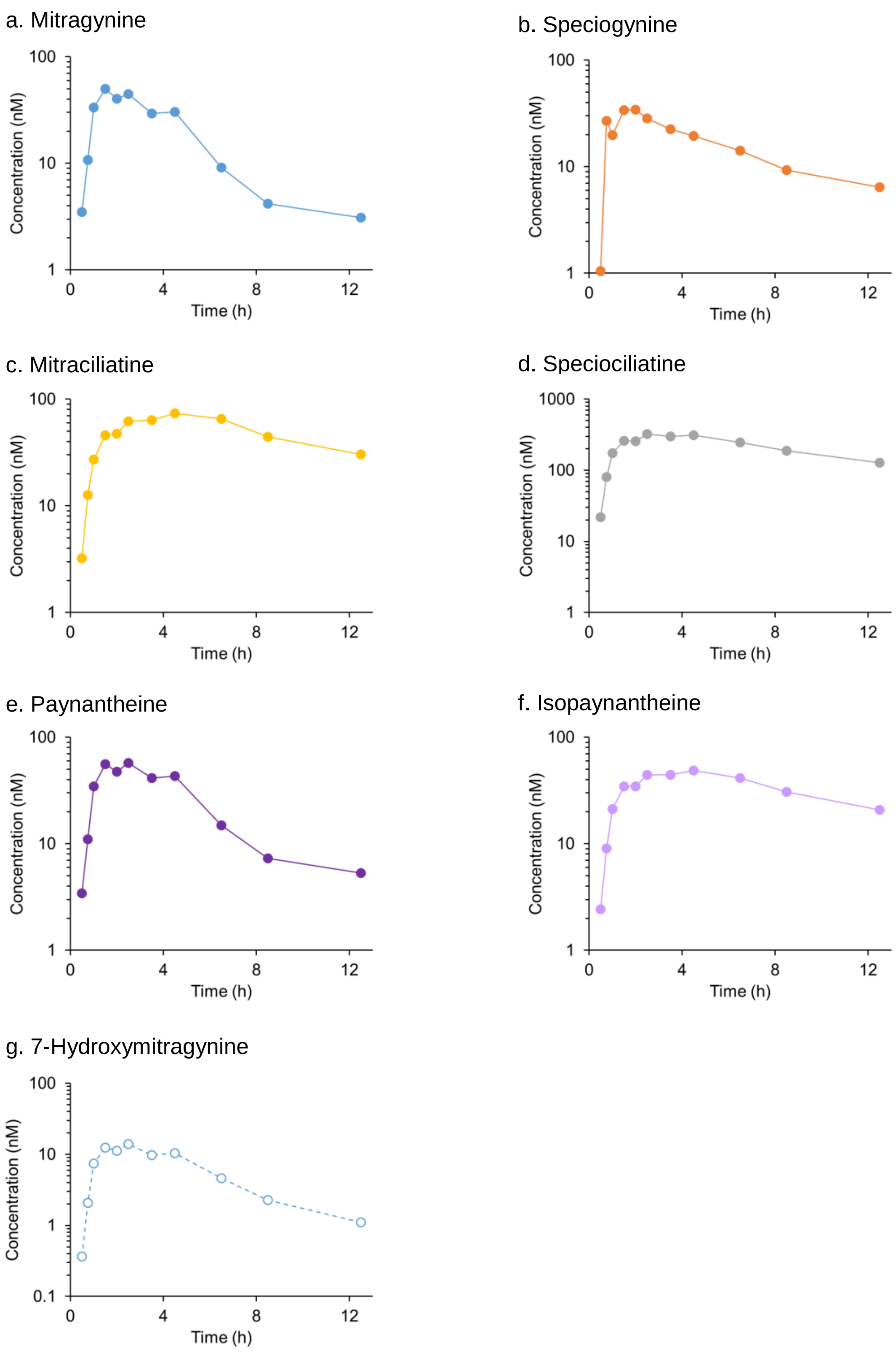

Figure S2. Representative concentration-time profiles from 0-12 hours for (a) mitragynine, (b) speciogynine, (c) mitraciliatine, (d) speciociliatine, (e) paynantheine, (f) isopaynantheine, and (g) 7-hydroxymitragynine in one participant, showing multiple peaks or gradations. These trends were observed in all participants.

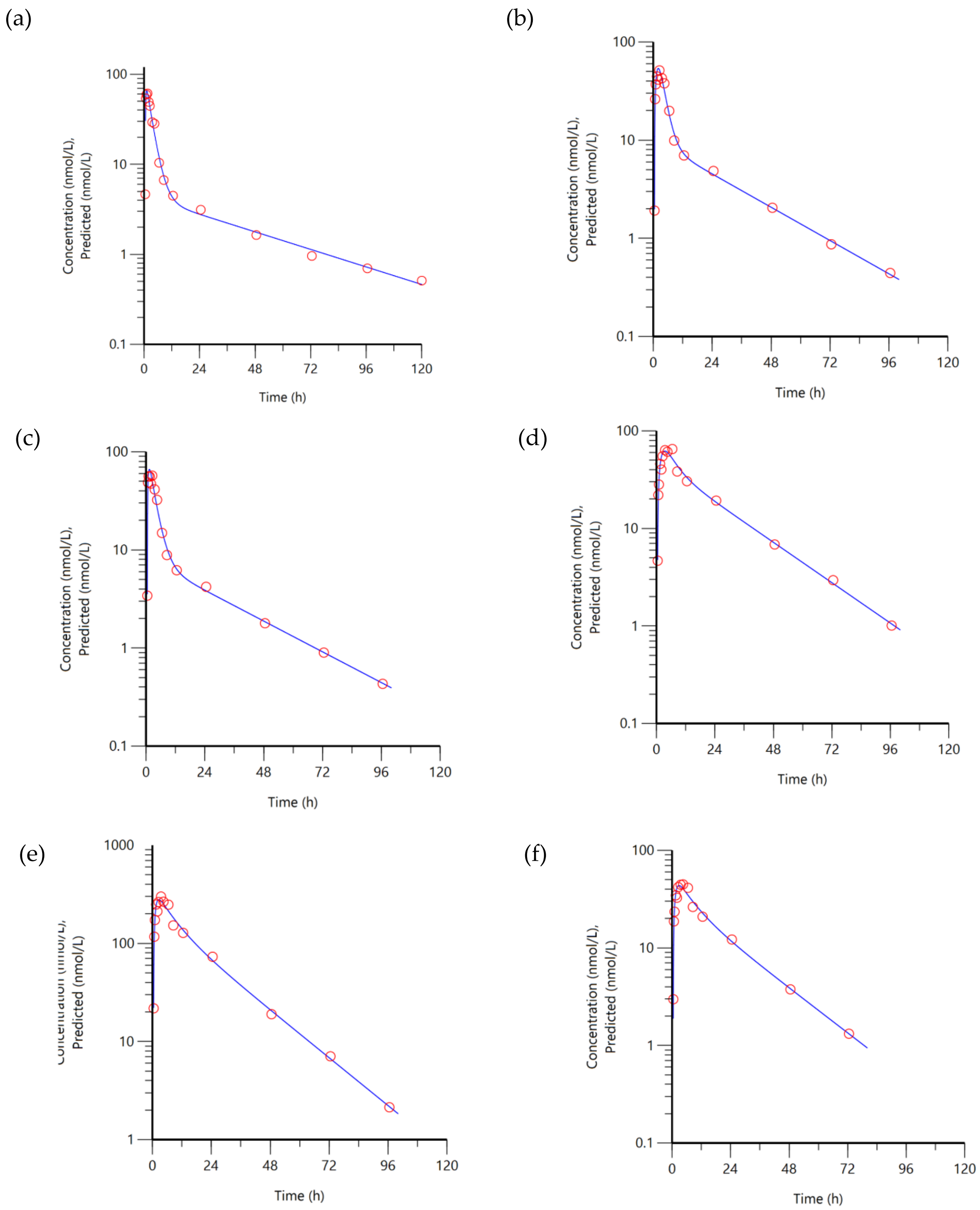

Figure S3. Red markers indicate observed median concentration-time profile of kratom alkaloids, from five participant who completed the study, including (a) mitragynine, (b) speciogynine, (c) paynantheine, (d) mitraciliatine, (e) speciociliatine, and (f) isopaynantheine. Blue solid lines indicate predicted concentration-time profile describing the observed data with a two-compartment model with first-order input and elimination rate, elimination from the central compartment, and a lag time.

(a) Participant A

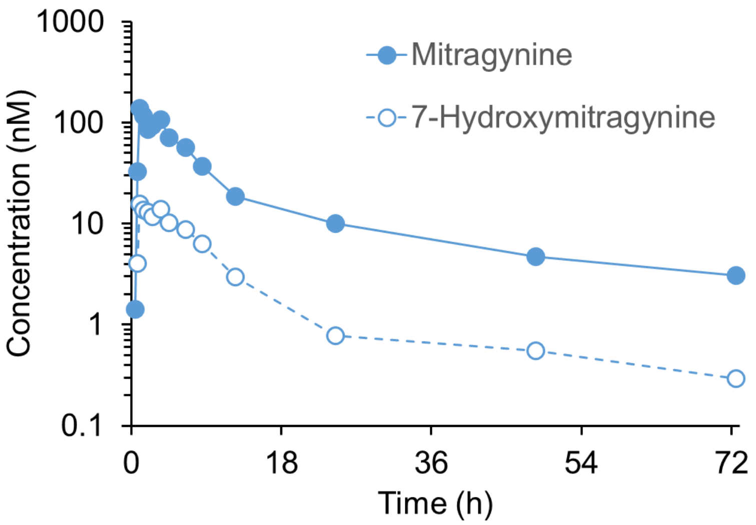

(b) Participant B

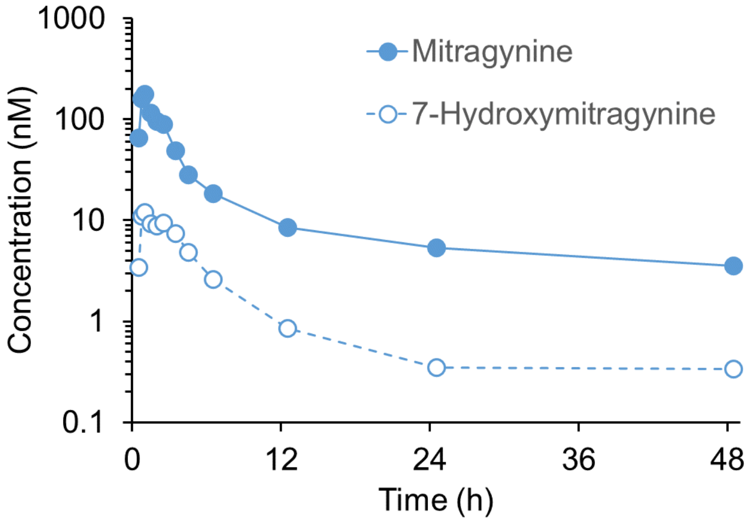

Figure S4. Plasma concentration-time profiles for the two participants in which 7-hydroxymitragynine was quantifiable beyond 24 hours. The parallel terminal portions of both profiles suggest 7-hydroxymitragynine follows formation rate-limited kinetics. Symbols indicate individual data points. (a). Participant A; (b) Participant B.

Table S1. Clinical study inclusion and exclusion criteria.

Inclusion:

- Aged from 18-55 years and healthy
- Not taking any medications (prescription and non-prescription) or dietary/herbal supplements known to modulate CYP3A, which converts mitragynine to 7-hydroxymitragynine
- Willing to abstain from consuming dietary/herbal supplements and citrus juices for several weeks
- Willing to abstain from consuming caffeinated beverages or other caffeine-containing products the evening before the inpatient visit
- Willing to abstain from consuming any alcoholic beverages for one day prior to the inpatient visit and each of the five outpatient visits
- Willing to use an acceptable method of contraception that does not include oral contraceptive pills or patches (such as abstinence, copper IUD, condom)
- Consume kratom intermittently (not regularly) and are willing to abstain for at least two weeks prior to beginning the study

Exclusion:

- Have never consumed kratom
- Any current major illness or chronic illness such as (but not limited to) kidney disease, hepatic disease, diabetes mellitus, hypertension, coronary artery disease, chronic obstructive pulmonary disease, cancer, or HIV/AIDS
- History of anemia or any other significant hematologic disorder
- History of seizure disorder
- History of drug or alcohol addiction or major psychiatric illness
- Women who are pregnant or nursing
- Need for chronic opioid pain medications
- Have a history of intolerance or allergy to kratom or opioids
- Used opioid pain medications within the last 3 weeks
- Taking concomitant medications, both prescription and non-prescription (including dietary supplements/ herbal products) known to modulate CYP3A

Table S2. Alkaloid content in commercial kratom product (K51).

Kratom alkaloid	Quantity in material (mg/g)[a]
mitragynine	19.48 ± 0.81[b]
speciogynine	3.18 ± 0.13
mitraciliatine	0.647 ± 0.035
speciociliatine	5.12 ± 0.26
paynantheine	5.86 ± 0.26
isopaynantheine	0.512 ± 0.010
7-hydroxymitragynine	< LOQ[c]

a.Quantity is denoted as mg of compound per g of dried kratom plant material as determined using methanolic extraction and subsequent analysis by UPLC-MS. b.Uncertainty calculated using standard deviation of triplicate extractions, each analyzed separately. c. Limit of quantitation (LOQ) for 7-hydroxymitragynine was 4.24 ng/mL; 7-hydroxymitragynine was detected in K51 but was below the LOQ.

The indole alkaloids (mitragynine, speciogynine, paynantheine, mitraciliatine, speciociliatine, and isopaynantheine) were isolated from commercial kratom powders and characterized in detail using proton (^1H) and carbon (^{13}C) nuclear magnetic resonance spectroscopy (NMR), high-resolution electrospray ionization mass spectrometry (HRESIMS), and electronic circular dichroism spectroscopy (ECD) data, as described previously,[1] and were all of a high purity (≥98%) as determined by ultra-high-performance liquid chromatography-ultraviolet spectroscopy (UHPLC-PDA) analysis. 7-Hydroxymitragynine was purchased from Cayman Chemical (Ann Arbor, MI). The purity and identity of this standard was verified by acquiring the ^1H NMR, ^{13}C NMR, HRESIMS, and ECD data, all of which were consistent with literature values.

The analytical method to quantify kratom alkaloids in commercial products was developed and validated following guidance from the Association of Official Analytical Collaboration (AOAC) International guidelines for single-laboratory validation of chemical methods for dietary supplements and botanicals.[2] Alkaloids in K51 were identified and quantified using HRESIMS by comparing retention time, accurate mass, and fragmentation patterns with the relevant standards. Alkaloid quantities (Table S1) were determined by plotting the area for the protonated molecular ion of the relevant standard versus concentration. Least-squares regression was conducted with $1/x^2$ weighting, and alkaloid concentrations were calculated by substituting peak area into the equation for the best fit line of the relevant standard. Alkaloid peak areas for the extract were within the linear portions of the relevant calibration curves.
